# Ataxia Telangiectasia Presenting as Cervical Dystonia

**DOI:** 10.7759/cureus.30723

**Published:** 2022-10-26

**Authors:** Priyal LNU, Vineet Sehgal, Saniya Kapila, Nihal Gulati, Lucky Bhalla Sehgal

**Affiliations:** 1 Neurology, Sehgal's Neuro and Child Care Centre, Amritsar, IND; 2 Neurology, Lady Hardinge Medical College, Delhi, IND; 3 Neurology, Amandeep Medicity, Amritsar, IND; 4 General Practice, Fortis Escorts Hospital, Amritsar, IND; 5 General Practice, Navpreet Hospital, Amritsar, IND; 6 Paediatrics, Sehgal's Neuro and Child Care Centre, Amritsar, IND

**Keywords:** rare variant, genetic variant, cerebellar-ataxia, atm mutation, focal dystonia

## Abstract

The ataxia telangiectasia mutant (ATM) protein is a sensor and signal transducer that amplifies and communicates signals of DNA damage further to the mediators of cell cycle arrest, apoptosis, and senescence (p16, p19, p21, BAX etc.) which is modified by the strength of the cellular stress. They are able to act as recognition and signaling proteins because of their kinase activity. Classic ataxia telangiectasia is associated with homozygous mutations of the ATM gene, the complete absence of its kinase activity and/or deleterious ATM gene mutations such as truncation/nonsense mutations, loss of function mutation, non-conservative substitutions, frameshift, and deletions. On the other hand, variant ataxia-telangiectasia (A-T) is associated with the presence of residual kinase activity. We report a six-year-old male patient who presented to us with abnormal neck movements as his initial complaint. ATM gene analysis showed a rare pathogenic variant of the ATM gene. The variant was a homozygous nonsense mutation in exon 2 of the ATM gene that resulted in the formation of a stop codon and premature truncation of the protein at codon 23 in exon 2 (p.Arg23Ter). In conclusion, we report a case of an unusual presentation of classic A-T. We should pursue a long-term follow-up and maintain a low threshold for performing pedigree analysis and genetic testing in pediatric patients with movement disorders. In resource-limited settings where kinase enzyme assays are not universally available to patients, web-based mutation prediction tools may be beneficial to predict the deleterious effects of the mutation.

## Introduction

Ataxia-telangiectasia (A-T) is a genetic disorder inherited in an autosomal recessive manner. Patients typically show symptoms during early childhood, puberty, and infrequently adulthood. It is characterized by respiratory complaints such as bronchitis, bronchiectasis, and sinusitis; neurological complaints such as poor muscle coordination (clumsiness); and movement disorders such as dystonia, choreoathetosis, myoclonus, and tremors [1]. General physical appearance is characterized by the presence of short stature, café au lait spots, progeric skin and hair changes, oculocutaneous telangiectasia, horizontal head thrusts due to difficulty in initiating horizontal saccades (both voluntary and reflex saccades are affected), ocular apraxia, diabetes mellitus, and features of glucose intolerance like acanthosis nigricans, hypogonadism, lymphocytopenias, predisposition to malignancy, hypoimmunoglobulinemia, and other immune defects [2-3].

The ATM gene codes for a kinase protein which acts as a recognition and signaling molecule for DNA damage from ionizing radiation. Depending on the level of stress, the ATM protein is capable of activating p16, p19, p21, BAX, and other mediators of cell cycle arrest, apoptosis, and senescence. Both apoptosis and senescence are different types of cell deaths, however, cells that die by senescence are still capable of producing mediators that promote carcinogenesis, whereas cells that die by apoptosis cease to function [4]. The latter refers to the mechanisms that cells employ to prevent the transfer of mutations to subsequent cell cycles [5-6]. This prevents cells from mutating into cancerous forms. Both classical and atypical A-T are the result of mutations in the ATM gene and both can result in cancer due to a dysfunction in the DNA damage signaling pathway. Classical cases of A-T have a life expectancy of 60 years, with the most common cause of death being respiratory complaints [7]. In the purview of neurology, the destruction of Purkinje cells and granule cells in the cerebellum, Betz cells in the cerebral cortex, and degeneration of white matter tracts such as the corticospinal tract and the spinocerebellar tract (impaired proprioception) appears to be the mechanism of neurological complaints (both motor and sensory) in ataxia telangiectasia. The loss of motor as well as sensory pathways manifests as ataxia. Some authors believe that the substantia nigra is involved, while others believe that deep gray matter affection, such as the basal ganglia, is to blame for movement problems such as dystonia and choreoathetosis seen in A-T.

## Case presentation

**Figure 1:**
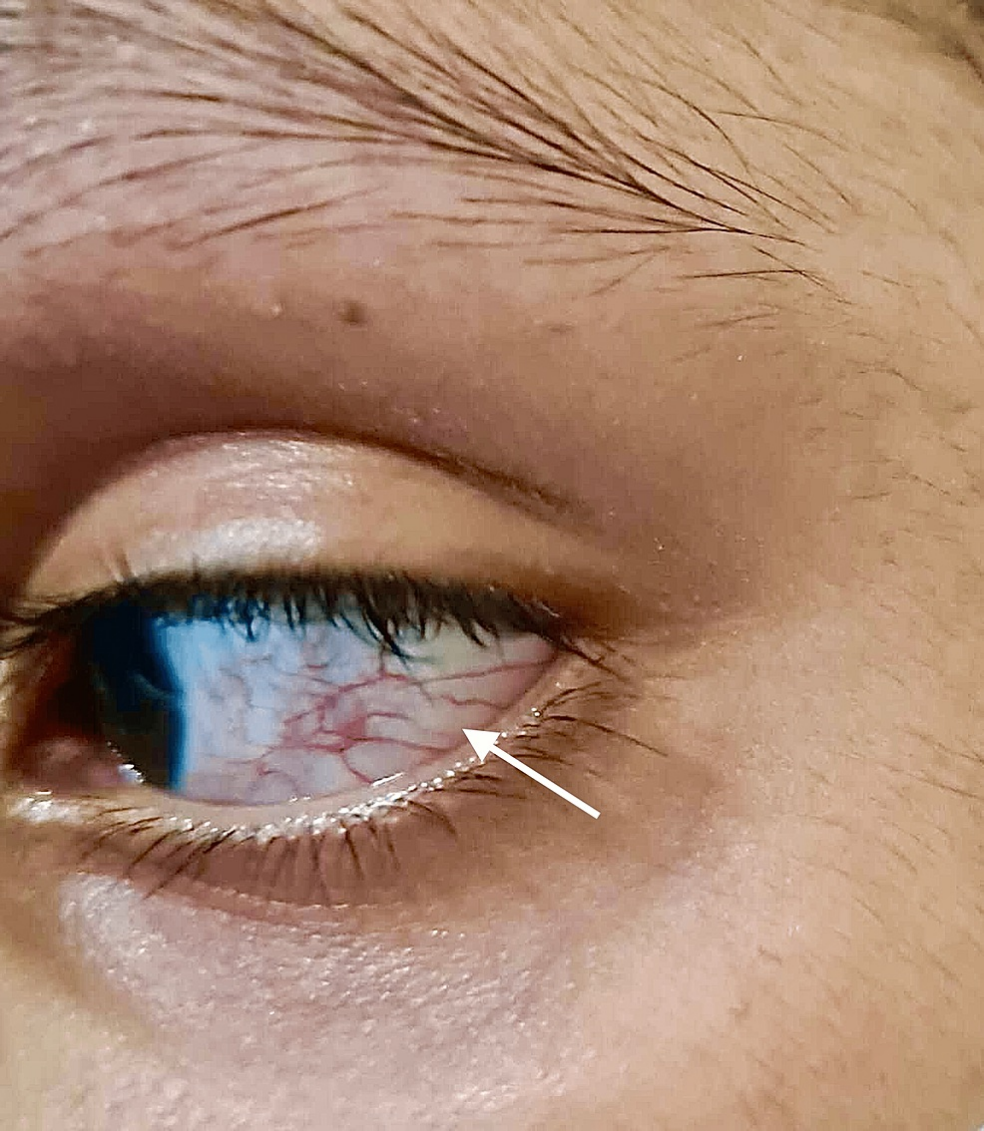
Six-year-old male with ataxia telangiectasia showing ocular telangiectasia

**Figure 2:**
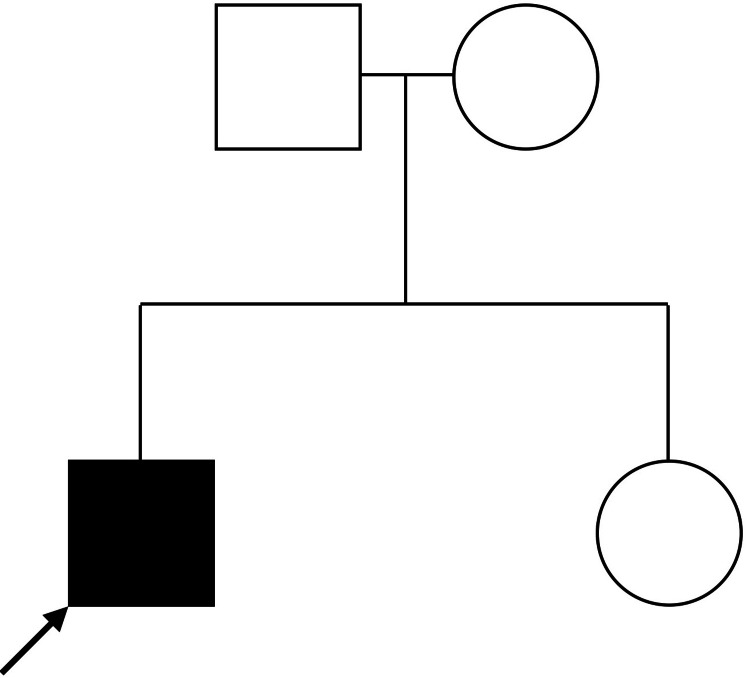
Pedigree chart based on the ATM gene analysis of the family of the six-year-old male patient suffering from classical ataxia telangiectasia (patient is depicted by the shaded box) The variant seen in the patient is a homozygous termination mutation at p.Arg23Ter in exon 2 of the ATM gene.

A six-year-old male patient presented to our neurology outpatient department with complaints of abnormal neck movements that had been worsening for six months, after which he began to experience balance issues while walking, gradually progressing to tremulousness in his hands and feet. He constantly splattered food on his clothes, had difficulty dressing and undressing by himself, playing with his friends, and walking to the bathroom without support. He had been suffering from recurrent bouts of bronchitis and eye redness for the past year (Figure 1). He had no history of similar complaints in his family (Figure 2). There were no reports of seizure-like behavior. There was no history of prior brain neurosurgery or brain trauma. There was no history of sinusitis, bronchiectasis, or hypogonadism.

The patient was afebrile, normotensive, and had a heart rate of 96 beats per minute, based on a general physical examination. The patient appeared to be of normal height for his age. He had café au lait spots all over his body and no other signs of abnormal skin or hair changes. There were no signs of glucose intolerance in the patient such as acanthosis nigricans, skin thickening, blistering, or discoloration.

The neurologic findings such as poor motor coordination with activity, dystonic neck tremors, abnormal heel-to-shin test, dysdiadochokinesia, past pointing, and impaired finger-to-nose test were positive in the patient. The rest of the neurological tests, including cranial nerves, motor examination, power assessment, reflexes, and sensory system were normal. There was no other hypokinetic or hyperkinetic movement such as myoclonus or choreoathetosis. There was no dystonia observed in other parts of the body. The strength was normal, and all four limb deep tendon reflexes were within normal limits; there was no abnormality of mood, thought, or speech content. In terms of loudness, rate, pitch, prosody, and articulation, the speech was normal. The patient was able to establish a good rapport with us.

As shown in the image, the patient’s conjunctiva had telangiectasia (Figure 1) [8]. The patient had no strabismus or fixation of gaze nystagmus on ocular examination, but smooth pursuit movements were impaired and saccades were hypometric. The remainder of the ocular examination was unremarkable.

On laboratory examination of the blood, the patient had lymphocytopenia and high levels of alpha-fetoprotein (AFP) of 180 nanograms per milliliter. A blood test ruled out diabetes mellitus. Vitamin E level was elevated at 12.3 micrograms per milliliter. Total IgA was less than 0.265 gram per liter (g/L), total IgG was 11.50 g/L, and total IgM was 1.36 g/L, which were all within normal limits. The level of kinase activity could not be determined due to a lack of kinase enzyme assays and western blot testing at our location.

ATM gene analysis showed a pathogenic variant of the ATM gene of the above-mentioned phenotype (cervical dystonic tremors, gait instability, limb ataxia, conjunctival telangiectasia, abnormal smooth pursuit movements of the eyes, and elevated AFP). The variant was a homozygous nonsense mutation in exon 2 of the ATM gene (locus of mutation was at chr11:g.108098418C>T; Depth: 84x) that resulted in a stop codon and premature truncation of the protein at codon 23 (p.Arg23Ter; ENST00000278616.4). The MutationTaster2, a web-based application for determining the disease-causing potential of mutations, predicted that the mutation was deleterious (Table 1).

**Table 1:** The genetic test results of the six-year-old male patient with classical A-T The coverage of the ATM gene is 100% in this genetic analysis. The genetic test results are reported based on the recommendations of the American College of Medical Genetics. MutationTaster2, a web-based application for determining the disease-causing potential of mutations, predicted that the mutation was harmful.

Gene (Transcript)	Location	Variant	Zygosity	Disease (OMIM)	Inheritance	Classification
ATM (+) (ENST00000278616.4)	Exon 2	c.67C>T (p.Arg23Ter)	Homozygous	Ataxia-telangiectasia	Autosomal recessive	Pathogenic

The genetic testing of the ATM gene of the patient's parents and sister yielded normal results (Figure 2). The cerebellum was normal as seen on the MRI of the brain.

On history and physical examination, dopa-responsive dystonia, primary torsion dystonia, and myoclonic dystonia were ruled out [9-10]. Concerning this case report, we got written permission from the patient’s parents to share his information and image.

The patient was diagnosed with dystonic tremors of the neck, gait ataxia, and appendicular ataxia cerebellar type, conjunctival telangiectasia, and recurrent bronchitis due to underlying classical ataxia telangiectasia. He was treated with a multi-disciplinary approach that included exercise, gait training, and physical therapy for his persistent ataxia, cancer screening with an oncologist, and lymphocytopenia treatment with a hematologist.

## Discussion

The worldwide prevalence of ataxia telangiectasia is between one in 40,000 and 100,000. The p.Arg23Ter variant has a minor allele frequency of 0.002% in the genomAD database. The Online Mendelian Inheritance in Man (OMIM) code for the phenotype is OMIM#208900. Ataxia-telangiectasia is usually caused by homozygous or compound heterozygous mutations of the ATM gene (OMIM* 607585), which leads to a reduced or complete absence of kinase enzyme activity. The level of kinase activity is a major determinant of the clinical phenotype of the patients. Most pathogenic or potentially pathogenic mutations in ATM gene exome sequences are non-conservative missense mutations, deletions, frameshift mutations, or putative loss of function mutations. Only a handful of silent mutations are known to be pathogenic. Consequently, it is possible to grossly infer a genotype-phenotype association even without kinase enzyme activity levels. This correlation is achieved by classifying the patient into one of two distinct A-T categories (classical and atypical) based on the genetic analysis's results and prediction of deleterious mutations (non-conservative substitution mutations, nonsense mutations, deletions, frameshift mutations, or putative loss of function mutations being the typical pathogenic forms according to ClinVar classification system) [11]. 

Patients with a homozygous mutation usually have some form of neurological complaint [1,5]. The atypical A-T has milder clinical features and cerebellar ataxia, oculocutaneous telangiectasia, and oculomotor apraxia are uncommon in atypical A-T [3]. The mutations in atypical A-T segregate within families, and the affected members of the family as a whole may present with movement problems rather than ataxia as the major hallmark of the condition. Dystonia occurs more often in atypical A-T than in classic A-T. It usually appears during childhood or adolescence and tends to affect multiple systems over time [12-15]. Depending on the time of presentation and level of kinase activity, dystonia may be a sole manifestation or may present with other movement disorders [7-9]. Our case report describes a rare truncation mutation of the ATM gene, identified as a deleterious mutation by the Mutationtaster2 web program employed for estimating the severity of mutations. The test result is consistent with classic ataxia telangiectasia. On the contrary, our patient's clinical presentation of dystonic neck tremors is well documented in the literature on both classical A-T and atypical A-T [16].

The pathogenesis of extrapyramidal movement disorders seen in A-T seems to be the destruction of Purkinje cells and granule cells in the cerebellum, the presence of Betz cells in the cerebral cortex, and degeneration of white matter tracts such as corticospinal tract, and spinocerebellar tracts leading to defective large sensory fibers such as those for proprioception all leading to neuropathy [17]. Spinal muscular atrophy-like presentations occur in these patients with interosseous muscular atrophy in the hands in combination with the early-onset dystonic posturing which leads to striking combined flexion-extension contractures of the fingers. Although intellectual disability is not a characteristic of A-T, some older patients suffer from significant short-term memory loss. The mutation observed in our case report is rare and cervical dystonic tremors are an unusual occurrence as primary complaints in A-T. Such a case has previously been reported in some cases of ataxia telangiectasia [16]. To assess the phenotypic variability of classical A-T, a large-scale investigation of the multitude of neuro-motor manifestations of A-T should be conducted.

## Conclusions

Dystonic neck tremors, as illustrated in this case report of a six-year-old boy, are seldom seen at the onset of classic A-T. The combination of a positive AFP screening and genetic testing for ATM mutation along with a history of dystonic neck tremors, conjunctival telangiectasia and recurrent bronchitis at the onset with progression into gait and limb ataxia, and abnormal smooth pursuit movements of the eyes, strongly suggested the diagnosis of classical A-T in our case. Genetic analysis alone cannot determine the pathogenicity of mutation. It needs confirmation by kinase enzyme assays or western blot analysis. In locations with limited resources, patients frequently lack access to western blotting and assays for measuring ATM kinase enzyme activity and the pathogenicity of the mutation should be supported by deleterious-mutation-prediction software. The degree of kinase enzyme activity corresponds with the clinical severity of the disease. 

Finally, this case highlights the importance of the need to conduct a large-scale study to learn about the multitude of neuro-motor manifestations of A-T. As these may be rare manifestations of otherwise common genetic disorders such as A-T, we should seek a long-term follow-up and maintain a low threshold for performing pedigree analysis and genetic testing during the initial encounter of these pediatric patients with movement complaints.
